# PD‐1 Inhibitor (Sintilimab) Combined With Chemotherapy as Neoadjuvant Therapy in Advanced Ovarian Cancer: A Case Report

**DOI:** 10.1002/ccr3.73598

**Published:** 2026-09-25

**Authors:** Wang Zhenyan, Wang Yan

**Affiliations:** ^1^ Department of Gynecology The Affiliated Hospital of Qingdao University Qingdao City Shandong Province China

**Keywords:** case report, neoadjuvant therapy, ovarian cancer, PD‐1 inhibitor, sintilimab

## Abstract

In a patient with advanced ovarian cancer showing limited response to paclitaxel–carboplatin, adding sintilimab was associated with R0 cytoreduction and sustained disease‐free status during olaparib–bevacizumab maintenance. This case provides a rationale for further investigation of PD‐1 blockade as neoadjuvant therapy in carefully selected patients with suboptimal response to platinum–taxane chemotherapy.

## 1 Introduction

Ovarian cancer is one of the most common and lethal malignancies of the female reproductive system, with the highest mortality rate among gynecologic cancers [1]. Due to the lack of specific early symptoms, approximately 80% of patients are diagnosed at an advanced stage, resulting in a poor prognosis [1]. Thus, achieving R0 resection remains the ultimate surgical goal for advanced ovarian cancer (AOC) [1]. This case report describes a patient with AOC who received neoadjuvant chemotherapy (NACT) consisting of the PD‐1 (programmed cell death protein 1) inhibitor sintilimab combined with paclitaxel and carboplatin, followed by optimal cytoreductive surgery that achieved R0 resection. Postoperative maintenance therapy with olaparib combined with bevacizumab has been administered to date, and the patient remains asymptomatic. The novelty of this case lies in the application of sintilimab combined with the aforementioned paclitaxel plus carboplatin as neoadjuvant therapy. This sequential regimen yielded a favorable tumor response and facilitated R0 interval cytoreductive surgery.

## 2 Case History

**FIGURE 1:**
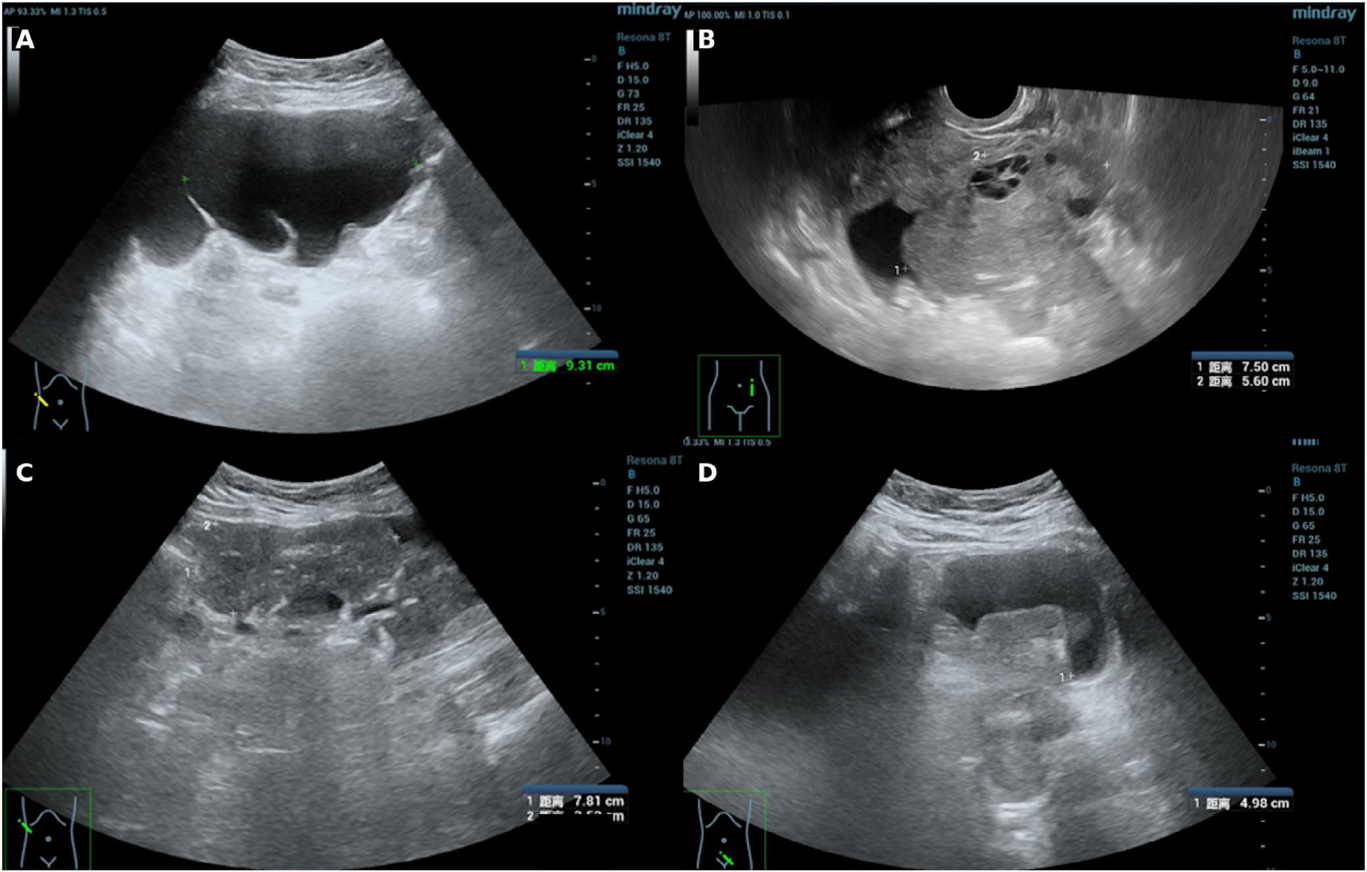
Ultrasound on August 22, 2024 (A–D). Pre‐treatment transvaginal ultrasound reveals complex bilateral adnexal masses, multiple hypoechoic lesions on the omentum and bowel serosa, and abundant abdominopelvic free fluid.

**FIGURE 2:**
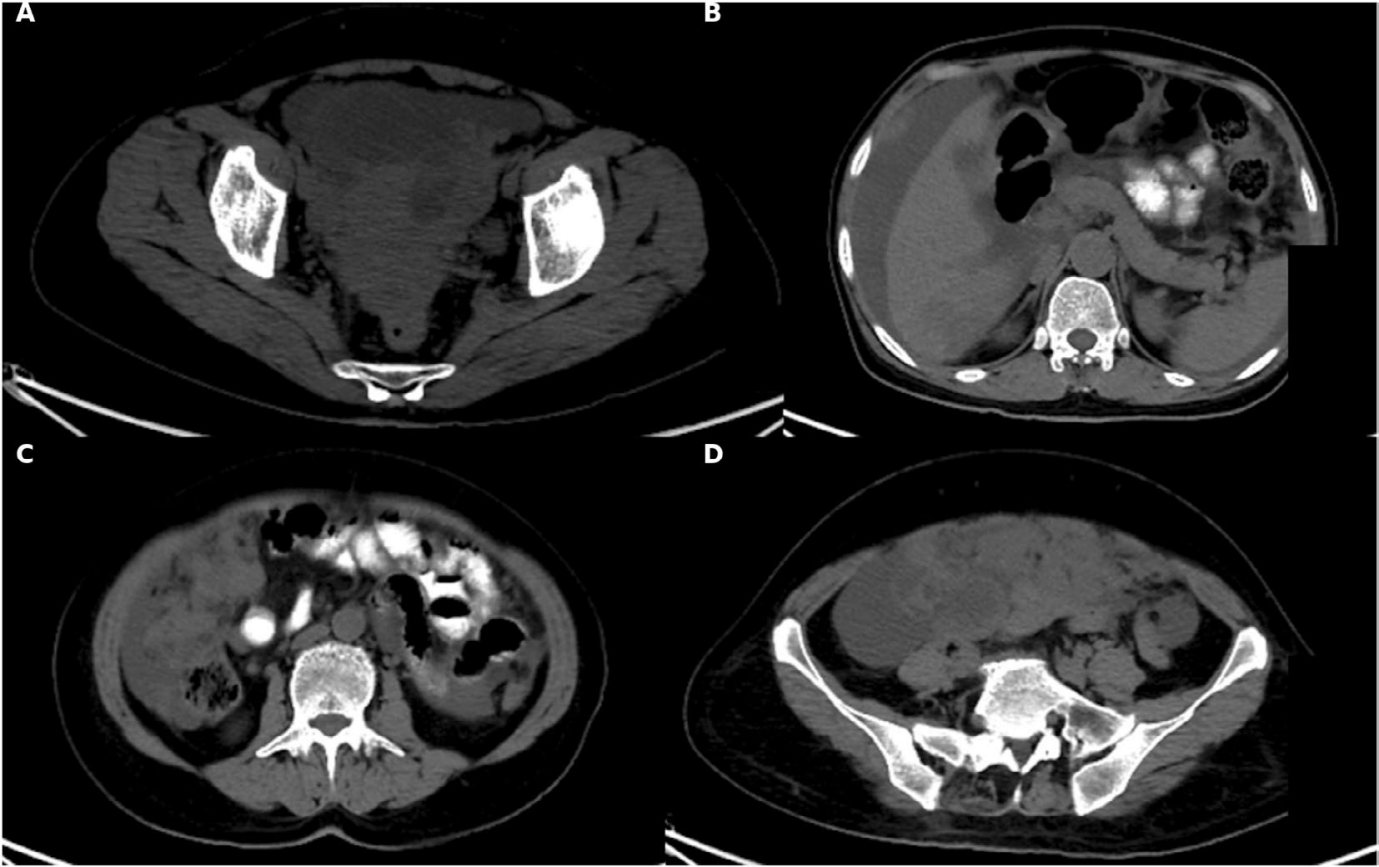
PET‐CT on August 22, 2024 (A–D). Baseline PET‐CT displays hypermetabolic bilateral adnexal lesions, diffuse peritoneal and omental implants, and massive ascites consistent with peritoneal carcinomatosis.

A 61‐year‐old woman presented to the Department of Gynecology on August 20, 2024, with progressive abdominal distension for 5 months. Physical examination revealed abdominal distension with positive shifting dullness. Laboratory tests showed elevated tumor markers: CA‐125: 562 U/mL; HE4: 303 pmol/L. Transvaginal ultrasonography revealed complex masses in both adnexal areas (right: 9.2 × 9.3 × 6.5 cm; left: 7.5 × 7.1 × 5.6 cm), multiple hypoechoic masses on the surface of the omentum and parts of the bowel, the largest measuring 7.8 × 4.7 × 3.5 cm, and free fluid in the pelvic and abdominal cavities, measuring 5.0 cm in the deepest pelvic region. Lesions were measured in three orthogonal dimensions on longitudinal and transverse scans (Figure 1). PET‐CT demonstrated bilateral adnexal masses, peritoneal, mesenteric, and omental implants with hypermetabolism, and massive ascites, suggesting AOC with peritoneal carcinomatosis (Figure 2). Cytology of ascitic fluid indicated malignancy. Immunohistochemistry: CK7 (+), CK20 (−), WT‐1 (+), P53 (mutant type), Pax‐8 (+), P16 (partly +), Ki‐67 (~70%), CDX‐2 (−), TTF‐1 (−), GATA3 (−). From August 27 to October 29, 2024, the patient received three cycles of paclitaxel plus carboplatin (PT regimen) with no significant tumor regression on imaging (per Response Evaluation Criteria in Solid Tumors, RECIST v1.1).

**FIGURE 3:**
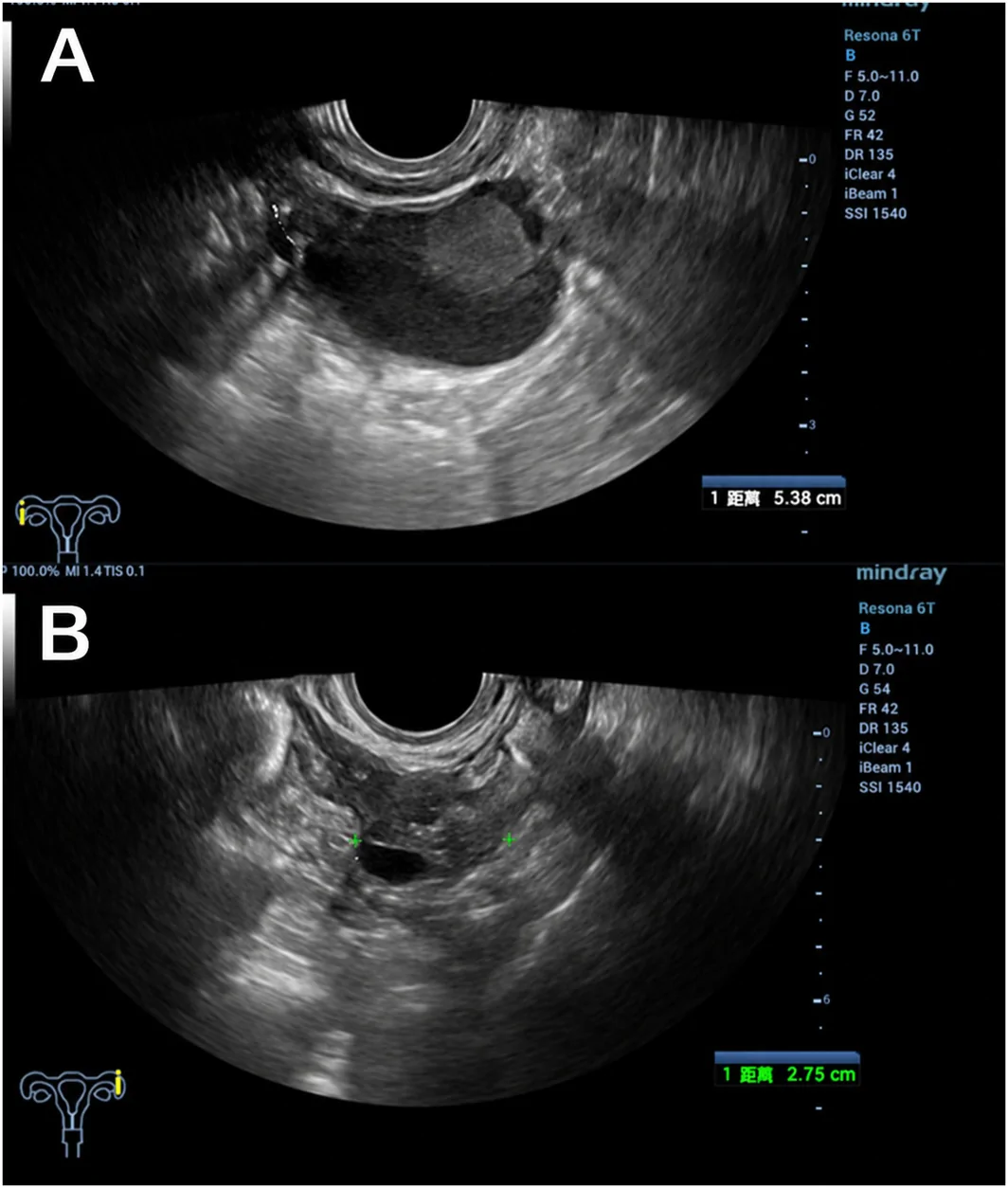
Ultrasound on February 14, 2025 (A, B). Post‐neoadjuvant ultrasound after sintilimab plus paclitaxel–carboplatin chemotherapy shows marked reduction of bilateral adnexal masses and disappearance of pelvic effusion.

A multidisciplinary team (MDT) comprising gynecologic oncology, medical oncology, radiology, and surgery concluded that the patient had shown a limited response to PT chemotherapy and that upfront surgery was unlikely to achieve R0 resection. It was therefore recommended to initiate chemotherapy combined with immunotherapy. Bevacizumab was withheld preoperatively due to the anticipated risks of intraoperative bleeding and intestinal fistula associated with extensive cytoreductive surgery and intestinal lesion resection. From November 9, 2024, to February 1, 2025, the patient received four cycles of paclitaxel plus carboplatin combined with sintilimab. Follow‐up ultrasonography on February 14, 2025, showed marked reduction of the bilateral adnexal lesions, with the right adnexal mass decreasing to 5.4 × 4.9 × 3.7 cm and the left adnexal lesion to 2.8 × 2.0 × 1.2 cm. No obvious fluid collection was detected in the pouch of Douglas (rectouterine pouch; Figure 3).

On February 17, 2025, the patient underwent total hysterectomy with bilateral salpingo‐oophorectomy, pelvic and para‐aortic lymphadenectomy, presacral lymph node dissection, omentectomy, and excision of bowel surface nodules.

Intraoperatively, a 6 cm right ovarian mass and multiple omental nodules (largest 2 cm) were identified. R0 was achieved. Postoperative pathology revealed poorly differentiated serous adenocarcinoma of the left ovary; no residual tumor was identified in the right ovary, uterus, omentum, or lymph nodes (consistent with R0 resection). Postoperatively, from March 18 to April 29, 2025, the patient received three cycles of paclitaxel plus carboplatin plus bevacizumab. Genetic testing revealed wild‐type BRCA1/2 and HRD positivity, indicating potential sensitivity to PARP inhibitors. According to the Chinese Guidelines for Gynecologic Cancer (CGCG, 2025 Edition), maintenance therapy with olaparib (300 mg, twice daily) and bevacizumab was initiated.

At the most recent follow‐up on January 4, 2026 (10 months after surgery), CT imaging and tumor marker tests showed no evidence of recurrence. The patient remains clinically disease‐free, although longer follow‐up is required.

## 3 Methods

The differential diagnosis included primary epithelial ovarian cancer, primary peritoneal carcinoma, and metastatic gastrointestinal adenocarcinoma. Diagnostic evaluation included physical examination, serum tumor‐marker testing, transvaginal ultrasonography, PET‐CT, ascitic‐fluid cytology, immunohistochemistry, postoperative histopathology, and BRCA1/2 and homologous recombination deficiency testing. The distribution of the adnexal and peritoneal lesions and the Müllerian immunophenotype supported an ovarian primary.

Radiologic response was evaluated according to RECIST version 1.1. Treatment decisions were made through MDT review. The patient received paclitaxel plus carboplatin, followed by the addition of sintilimab because of the limited response to initial chemotherapy. After interval cytoreductive surgery, postoperative paclitaxel, carboplatin, and bevacizumab were administered, followed by olaparib plus bevacizumab maintenance therapy. Follow‐up included clinical assessment, serum tumor‐marker testing, and CT imaging.

## 4 Conclusions and Results

This single‐case experience indicates that sintilimab combined with paclitaxel plus carboplatin may represent a neoadjuvant strategy for carefully selected patients with advanced high‐grade serous ovarian cancer who demonstrate a suboptimal response to upfront platinum–taxane chemotherapy. Importantly, this observation is purely hypothesis‐generating; the patient's favorable short‐term oncologic outcome cannot be ascribed exclusively to sintilimab, given the combined therapeutic effects of chemotherapy, radical surgery, bevacizumab, and PARP inhibitor maintenance therapy. Extended follow‐up, expanded case series, and prospective clinical trials are necessary to define regimen efficacy, safety profiles, and optimal patient selection criteria.

## 5 Discussion

This case describes the use of sintilimab combined with paclitaxel plus carboplatin as neoadjuvant therapy for a patient with advanced high‐grade serous ovarian cancer, who initially achieved only a limited radiologic response to this first‐line chemotherapy regimen. PD‐1 is a coinhibitory receptor expressed on T cells that restrains autoimmunity via downregulation of immune effector activation [2]. Its ligand, PD‐L1, is expressed on tumor and immune cells within the tumor microenvironment [3]. The PD‐1/PD‐L1 interaction mediates immune evasion by tumors. Blocking this pathway restores cytotoxic T‐cell activity and enhances antitumor immunity [4]. Clinical evidence supporting immune checkpoint inhibitors for ovarian cancer remains limited relative to many other solid malignancies. In the KEYNOTE‐100 trial, pembrolizumab monotherapy demonstrated only modest antitumor activity in recurrent ovarian cancer [5]. Similarly, the Phase III IMagyn050/GOG 3015/ENGOT‐OV39 trial failed to yield a meaningful progression‐free survival benefit when atezolizumab was added to frontline chemotherapy plus bevacizumab in the overall cohort of patients with newly diagnosed Stage III or IV ovarian cancer [6]. These findings demonstrate that checkpoint blockade cannot be presumed universally effective for ovarian cancer; its clinical utility hinges on treatment setting, combination regimen, tumor biology, and patient selection. NACT is an important strategy in the multidisciplinary treatment of ovarian cancer. NACT can reduce tumor burden and facilitate complete cytoreductive surgery (R0 resection). In our case, the patient showed a limited response to PT chemotherapy. After sintilimab was added to the PT backbone regimen, the tumor burden regressed markedly, permitting R0 interval cytoreduction. Sintilimab, a PD‐1 inhibitor independently developed in China, has been investigated across multiple hematologic and solid malignancies [7]. Nevertheless, data supporting sintilimab as neoadjuvant therapy for ovarian cancer remain limited. Evidence from other malignancies (e.g., triple‐negative breast cancer, non‐small‐cell lung cancer) indicates that early delivery of PD‐1/PD‐L1 immune checkpoint inhibitors improves clinical outcomes [8, 9], likely driven by greater preoperative tumor antigen release and robust CD8+ T‐cell tumor infiltration [10, 11]. Compared with adjuvant therapy, neoadjuvant checkpoint blockade may induce a stronger and broader tumor‐specific immune response [9], enhancing immune surveillance and eradicating micrometastases [12]. Bevacizumab, a well‐established antiangiogenic agent for AOC, may also be incorporated into neoadjuvant regimens for carefully selected patients [13]. Available evidence suggests that neoadjuvant bevacizumab does not necessarily worsen perioperative outcomes when an adequate preoperative washout interval is maintained, and an interval of approximately 6–8 weeks before major surgery is commonly recommended because of its long half‐life and potential effects on wound healing [13, 14]. In this patient, however, preoperative bevacizumab was not selected because the MDT anticipated extensive interval cytoreduction with excision of bowel‐surface lesions and possible bowel resection, making the consequences of impaired wound healing, bleeding, gastrointestinal perforation, or fistula formation particularly relevant. Although an adequate washout interval reduces perioperative concern, it does not eliminate these risks, and the MDT therefore favored sintilimab as an individualized neoadjuvant adjunct after the limited response to PT chemotherapy. Thus, bevacizumab with an appropriate washout interval would also have been a reasonable alternative strategy [13, 14], and our treatment choice should not be interpreted as evidence that immunotherapy is superior to bevacizumab in the neoadjuvant ovarian cancer setting. A further rationale for adding immunotherapy as a neoadjuvant adjunct stems from the potential synergistic antitumor interplay between PD‐1 blockade and platinum–taxane chemotherapy. Chemotherapy can potentiate immunotherapy by releasing tumor antigens, reversing immunosuppression in the tumor microenvironment, and promoting effector T‐cell infiltration [15]. Consistent with this mechanistic framework, the sintilimab–chemotherapy combination in our patient yielded substantial tumor regression and allowed optimal interval cytoreductive surgery. Notably, the patient carried wild‐type BRCA1/2 yet tested HRD‐positive; she attained clinical disease‐free status following postoperative therapy and sustained disease‐free status throughout maintenance treatment. This observation should be interpreted cautiously because postoperative bevacizumab and olaparib may have contributed substantially to the disease‐free status.

Although preoperative PET‐CT showed no suspicious nodal involvement, we performed pelvic, para‐aortic, and presacral lymphadenectomy; final postoperative pathology confirmed that all resected lymph nodes were negative for metastasis. Current randomized controlled trial (RCT) evidence demonstrates that systematic lymphadenectomy fails to confer a survival benefit for patients with AOC who attain macroscopic complete resection and have clinically negative lymph nodes; conversely, it may raise perioperative complication rates [16]. Nevertheless, high‐grade serous ovarian carcinoma carries a substantial risk of occult micrometastatic lymphatic spread, even without radiologic lymphadenopathy. Considering the patient's advanced tumor stage, we performed pelvic, para‐aortic, and presacral lymphadenectomy with the intent of removing potential microscopic nodal disease. This surgical decision balanced published trial evidence against the tumor's individual biologic features, reinforcing the value of personalized operative planning for AOC.

## Author Contributions

**Wang Zhenyan:** conceptualization, data curation, formal analysis, investigation, methodology, project administration, resources, supervision, writing – original draft, writing – review and editing. **Wang Yan:** data curation, investigation, project administration, writing – review and editing.

## Funding

The authors have nothing to report.

## Consent

Written informed consent was obtained from the patient for publication of this case report and the accompanying images.

## Conflicts of Interest

The authors declare no conflicts of interest.

## Data Availability

The data that support the findings of this case report are available from the corresponding author upon reasonable request. Relevant de‐identified data will be made available to the journal and reviewers for verification if required, subject to patient confidentiality and applicable privacy requirements.
